# Q&A: Primary generalized glucocorticoid resistance

**DOI:** 10.1186/1741-7015-9-27

**Published:** 2011-03-23

**Authors:** George Chrousos

**Affiliations:** 1 Department of Pediatrics, National University of Athens, Mikras Asias 75, 115 27, Athens, Greece

## What is 'Primary Generalized Glucocorticoid Resistance' (PGGR)?

PGGR means that all tissues in an organism have decreased sensitivity or "resistance" to the natural glucocorticoid, in us humans, cortisol.

## What biological processes underlie this "resistance" to cortisol?

**Figure 1:**
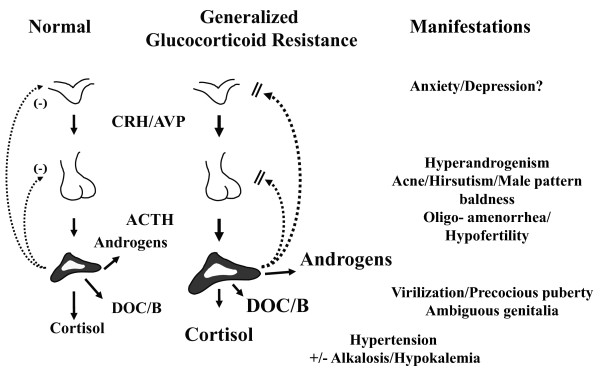
**Pathophysiologic Mechanisms of Familial or Sporadic Generalized Glucocorticoid Resistance (Chrousos) Syndrome**. In this syndrome, hypothalamic corticotropin-releasing hormone (CRH) and arginine-vasopressin (AVP) and pituitary adrenocorticotropic hormone (ACTH) are hypersecreted to compensate for the target tissue sensitivity defect, because of decreased glucocorticoid negative feedback. In response, the adrenal cortices hypertrophy and hypersecrete cortisol, adrenal androgens and steroid precursors, such as deoxy-corticosterone (DOC) and corticosterone (B) with mineralocorticod activity. The increased adrenal androgens may cause acne, hirsutism, male pattern baldness, oligo-amenorrhea and hypofertility in women and precocious puberty and ambiguous genitalia in children. The increased cortisol, DOC and B can cause hypertension with or without concurrent hypokalemic alkalosis. The increased hypothalamic CRH and AVP may cause anxiety and/or depression. The increased levels of ACTH may stimulate growth of adrenal rests in the testes of male patients causing oligospermia and infertility. (Modified from Chrousos GP, Detera-Wadleigh S, Karl M: **Syndromes of Glucocorticoid Resistance**. *Ann Int Med *1993, **119**:1113-1124).

Because this resistance includes the hypothalamic-pituitary-adrenal (HPA) axis negative feedback regulatory centers in the brain and pituitary gland, this axis is activated to "overcome" or compensate for the glucocorticoid action defect (Figure 1). The production of hypothalamic corticotropin-releasing hormone (CRH), arginine-vasopressin (AVP) and pituitary adrenocorticotropic hormone (ACTH) increase and the adrenal cortices hyperfunction, secreting large amounts of cortisol, adrenal steroid precursors and adrenal androgens. The adrenal steroid precursors include deoxy-corticosterone and corticosterone, both of which, as well as cortisol, have sodium-retaining activity through the mineralocorticoid receptor, which is normal in patients with cortisol resistance. Thus, the patient tissues are exposed to elevated levels of adrenal androgens and mineralocorticoids, which result in manifestations of hyperandrogenism and hypermineralocorticoidism.

## What are these manifestations of hyperandrogenism and hypermineralocorticoidism?

These manifestations include acne, hirsutism, oligo-amenorrhea, male-pattern baldness, and hypofertility in women and virilization in children, and hypertension and alkalosis with or without hypokalemia in both sexes, respectively.

## And are there any non-hyperandrogenism and non-hypermineralocorticoidism-related manifestations linked to PGGR?

Generally, there are no manifestations of glucocorticoid excess (Cushing syndrome) or deficiency (Addison disease), with the exception of chronic fatigue in some patients and profound hypoglycemia in a recently reported infant with complete glucocorticoid resistance. Similarly, there are no concretely proven manifestations expected from the increases of hypothalamic CRH, such as anxiety and depression.

## What is the etiology of PGGR?

The etiology of Familial or Sporadic Primary Generalized Glucocorticoid Resistance in most reported cases has been ascribed to molecular defects of the glucocorticoid receptor gene, however, one would expect manifestations of the syndrome in other faults of the glucocorticoid signaling system. Interestingly, there may be abnormalities in factors shared between the glucocorticoid signaling system and other cellular pathways, causing syndromes with both overlapping and distinct manifestations. For instance, defects in shared transcriptional co-activators might result in manifestations not only of glucocorticoid but also of other steroid hormone resistance states. In the early 1980s, my colleagues and I discovered that all New World primates compared to prosimian species or Old World primates have a fully compensated "pan-steroid" and sterol (Vitamin D) hormone resistance. We concluded then that there must be a nodal molecule(s) shared by all steroid/sterol hormone action networks in the cells of these animals. The identity of this molecule(s) remains unknown to this day. Whether this pan-steroid/sterol resistance conferred some selective advantage or whether it was the result of tolerated genetic drift also remains an enigma.

## How large an impact has the discovery and the subsequent clarification of the underlying pathophysiology of PGGR Syndrome had on the biomedical community as a whole?

The implications of the discovery of the PGGR Syndrome and the elucidation of its pathophysiologic mechanisms sensitized the biomedical community to expect and look for glucocorticoid signaling involvement in the pathophysiology of a large number of rare and common disorders, including autoimmune, allergic and behavioral disorders and the scourge of our modern aging societies, the "chronic noncommunicable diseases", such as obesity, hypertension, metabolic syndrome, diabetes mellitus type 2, cardiovascular and neurodegenerative diseases, and major depression. This realization occurred by making the usual logical extrapolations: (1) A severe disorder may have milder, even subclinical (*formes frustes*) forms; (2) a hormone resistance syndrome may have its mirror image, a hormone hypersensitivity syndrome; and, (3) a generalized resistance or hypersensitivity syndrome may have tissue-specific counterparts.

Indeed, over the last 30 years, we have recognized extremely mild generalized glucocorticoid resistance or hypersensitivity in the general population, which results from polymorphisms of the glucocorticoid receptor gene and which makes a difference in the longevity, and metabolic syndrome and depressive manifestations of the people who carry them. Also, a clear-cut rare case of generalized glucocorticoid hypersensitivity syndrome that resulted from an activating mutation of the glucocorticoid receptor gene was published. And, finally, we have several examples of tissue-specific glucocorticoid resistance and hypersensitivity in several immune, autoimmune/inflammatory and dysmetabolic states, including hematologic malignancies, AIDS, rheumatoid arthritis and visceral obesity/insulin resistance.

## A recent paper (Bouligand et al., *PLoS One*. 2010 22:5:e13563), proposes that glucocorticoid receptor haploinsufficiency compromises glucocorticoid sensitivity and may represent a novel genetic cause of subclinical hypercortisolism. How do the results of this paper impact on the understanding of this disease?

When I first read this paper I was surprised to find out that the authors did not refer to the first family that presented with familial glucocorticoid resistance because of haploinsufficiency of the glucocorticoid receptor gene, published by Karl *et al*. in 1993 (*J Clin Endocrinol Metab*. 1993 **76**:683-689). It is apparent that having 50% of the normal glucocorticoid receptor may cause primary glucocorticoid resistance manifestations, including hyperandrogenism, as in the human family, or hypermineralocorticoidism, as in the animal model. Adrenocortical hyperplasia is expected in primary glucocorticoid resistance as a result of the hyperstimulation and hyperfunctioning of the adrenal cortices.

## What are the current treatment strategies for PGGR?

The first patient with the disorder, and since then, many patients with PGGR have been treated successfully with conventionally high doses of synthetic glucocorticoids that have no inherent mineralocorticoid activity, such as dexamethasone, at clinically and biochemically titrated doses. Such medications suppress ACTH secretion and adrenocortical function without stimulating the mineralocorticoid receptor. Thus, hyperandrogenism and hypermineralocorticoidism are corrected, while glucocorticoid actions at the target tissues remain compensated.

## So is there a need for novel treatments for this condition?

Treatment with dexamethasone remains a relatively easy and practical approach. Of course, correcting the actual defect of the glucocorticoid receptor or any other molecule involved in the pathogenesis of the syndrome would be more natural, but I expect that this will happen when gene therapy technologies become available in the future.

## What are the difficulties encountered in forming a diagnosis of this disease given its fairly low prevalence rate?

Once we suspect the diagnosis, the evaluation is straightforward. In my experience, a patient with early Cushing syndrome may be referred because of a discrepancy between a mild clinical phenotype and high biochemical indices of hypercortisolism. The known prevalence of the condition is low, however, in reality, there are no systematic studies of large series of patients with hyperandrogenism or hypertension, two relatively common states, in whom cortisol secretion and its responsiveness to dexamethasone were evaluated.

## What prospects are there for the formation of novel tools to aid in the diagnosis of this condition?

We have developed accurate and sensitive clinical and laboratory methods to diagnose sporadic or familial primary glucocorticoid resistance. We also have at our disposal quite sophisticated tools to examine and define the molecular etiology of the disorder in the patients in whom known components of the glucocorticoid signaling system are defective. This knowledge will include defining and understanding not only the genetics but also the epigenetics of this system and their overall effect on the phenotype of the patient.

## Is this disease a good example in case of why we should be looking to expedite a move to more personalized medicine based on a patient's genetic profile?

Indeed it is. The glucocorticoid signaling system is involved in a major fashion in human physiology and pathology and knowing details about its activity will be part of the personalized genotype and "epigenotype" of an individual and his or her risk for developing certain diseases.

## A recent review by your long time collaborators Evangelia Charmandari and Tomoshige Kino (Charmandari and Kino, *Eur J Clin Invest *2010 40:932-942) has proposed renaming PGGR as 'Chrousos syndrome' in recognition of your ground-breaking work into this field. What are your feelings after this suggestion?

Obviously, I am greatly honored and thankful. Over my career, I have had the privilege of working with fantastic mentors, colleagues and students. Their trust, friendship, and continuing support have been a source of inspiration for me. I am proud of the over 60 physicians and scientists that trained in my laboratory and pursued successful, independent careers in Academia and industry. I see this proposal as a manifestation of appreciation and I am grateful.

## Given your long history and impressive work in this field, what, in your opinion, are the key breakthroughs made in understanding more about this syndrome?

The key breakthroughs in this syndrome are its discovery, the dexamethasone binding studies that pinpointed the defect to the glucocorticoid receptor, the understanding of its macropathophysiology and conceiving the principle of its therapy, the identification of the molecular defects, the conceptual extrapolation to glucocorticoid hypersensitivity states, the discovery of polymorphisms of the receptor and their epidemiologic significance, and the conceptual extrapolation and search for mild forms and tissue-specific pathologies.

## What do you think are the future directions of research into this field?

The new directions include: (1) finding the true prevalence of the syndrome in hyperandrogenism, hypertension and other conditions in which glucocorticoid resistance could be etiologically relevant; (2) exploring the interactions of the glucocorticoid signaling system and other cellular signaling pathways and their combined effect on human pathology; (3) looking further into the New World primates for nodal molecules of multiple networks related to steroid and sterol (Vitamin D) hormone actions; (4) exploring the tissue-specific changes of the glucocorticoid signaling system resulting in behavioral pathology. Tissue-specific and conditional knock-out and transgenic animals are key in this endeavor, however, soon, with the availability of sensitive and dynamic imaging methods, it will be hopefully possible to directly examine regions of the brain in which changes of the glucocorticoid signaling system could explain manifestations such as anxiety (amygdala), depression (dopaminergic reward system), and cognitive (frontal cortex) or memory dysfunction (hippocampus).

## Where can I find out more?

### Books

Chrousos GP, Loriaux DL, Lipsett MB, Eds. *Steroid Hormone Resistance: Mechanisms and Clinical Aspects*. New York: Plenum Press; 1986. In the Series: *Advances in Experimental Medicine and Biology*, Vol. 196.

Chrousos GP, Olefsky JM, Samols E, Eds. *Hormone Resistance and Hypersensitivity States*. Philadelphia: Lippincott, Williams and Wilkins; 2002. In *Modern Endocrinology Series*.

Kino T, Charmandari E, Chrousos G, Eds. *Glucocorticoid Action: Basic and Clinical Implications*. Ann N Y Acad Sci, 2004, Vol 1024.

### Articles

Bouligand J, Delemer B, Hecart AC, Meduri G, Viengchareun S, Amazit L, Trabado S, Fève B, Guiochon-Mantel A, Young J, Lombès M: **Familial glucocorticoid receptor haploinsufficiency by non-sense mediated mRNA decay, adrenal hyperplasia and apparent mineralocorticoid excess**. *PLoS One*. 2010, **5**:e13563.

Charmandari E, Kino T: **Chrousos syndrome: a seminal report, a phylogenetic enigma and the clinical implications of glucocorticoid signalling changes**. *Eur J Clin Invest *2010, **40**:932-942.

Charmandari E, Ichijo T, Jubiz W, Baid S, Zachman K, Chrousos GP, Kino T: **A Novel Point Mutation in the Amino Terminal Domain of the Human Glucocorticoid Receptor (hGR) Gene Enhancing hGR-mediated Gene Expression**. *J Clin Endocrinol Metab *2008, **93**:4963-4968.

Chrousos GP, Detera-Wadleigh S, Karl M: **Syndromes of Glucocorticoid Resistance**. *Ann Int Med *1993, **119**:1113-1124.

Chrousos GP and Kino T: **Glucocorticoid Signaling in the Cell: Expanding Clinical Implications to Complex Human Behavioral and Somatic Disorders**. In: *Glucocorticoids and Mood: Clinical Manifestations, Risk Factors, and Molecular Mechanisms*. *Pro. NY Acad Sci *2009, **1179**:153-166.

Chrousos GP, Renquist D, Brandon D, Eil C, Pugeat M, Vigersky R, Cutler G., Loriaux DL, Lipsett MB: **Glucocorticoid Resistance and Primate Evolution: Receptor-mediated Mechanisms**. *Proc Natl Acad Sci *1982, **179**:2036-2040.

Karl M, Lamberts SW, Detera-Wadleigh SD, Encio IJ, Stratakis CA, Hurley DM, Accili D, Chrousos GP: **Familial glucocorticoid resistance caused by a splice site deletion in the human glucocorticoid receptor gene**. *J Clin Endocrinol Metab*. 1993, **76**:683-689.

